# Locus Minoris Resistentiae in Coccidioidomycosis: A Case Series

**DOI:** 10.1177/2324709619858110

**Published:** 2019-06-21

**Authors:** Namgyal Sherpa, Rushabh Shah, Brian Nordstrom, Christine Palmares, Arash Heidari, Royce Johnson

**Affiliations:** 1 Kern Medical Center, Bakersfield, CA, USA

**Keywords:** coccidioidomycosis, valley fever, San Joaquin fever, locus minoris resistentiae

## Abstract

Locus minoris resistentiae refers to decreased resistance in any internal organ or external body region, leaving it more vulnerable to disease processes than other regions. These changes, either congenital or acquired, alter the defense capacity. The concept of locus minoris resistentiae is widely accepted in the medical field and presents itself across specialties. Antecedent trauma is a known risk factor for hematogenous dissemination of infection; this also applies to coccidioidal species. In this article, we describe 2 patients who suffered from pulmonary coccidioidomycosis with subsequent trauma resulting in osseous dissemination to the site of injury.

## Introduction

Coccidioidomycosis infection occurs predominantly by inhalation with its primary infection typically unrecognized.^1^ Disseminating via lymphohematogenous spread from the initial pulmonary lesion, it can present with extrapulmonary manifestations, most commonly in subcutaneous soft tissue. Additional sites of dissemination include, but are not limited to, the skeleton, meninges of the brain, and spinal cord.^2-5^ However, there is no evident correlation between these coccidioidal lesions and their site of localization with the exception of a well-described medical phenomenon known as locus minoris resistentiae (LMR).^6^ LMR refers to an increased susceptibility to an insulting agent in a particular body region resulting in its localization. The vulnerability of the region can be either congenital or acquired, such as antecedent trauma in these cases.^7-9^ In this article, we describe 2 patients who suffered from pulmonary coccidioidomycosis with subsequent trauma, resulting in osseous dissemination to the site of injury.

## Case Presentation 1

The case is of an 18-year-old Filipino male resident of San Joaquin Valley with history of fever, body ache, fatigue, and dry cough 4 month prior to admission (PTA). The patient was a field worker who was previously healthy. These symptoms persisted for 3 weeks with new-onset generalized maculopapular rash on his hands, forearms, and shins. A primary care visit resulted in a diagnosis of viral infection and he received antihistamines. His rashes resolved 3 months PTA, with his cough and fatigue persisting.

**Figure 1.**
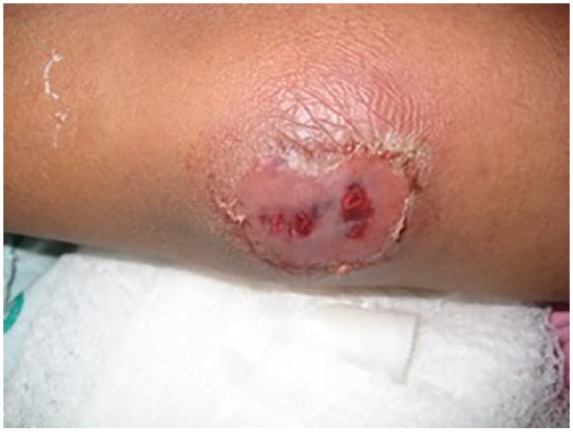
Right elbow on admission.

**Figure 2.**
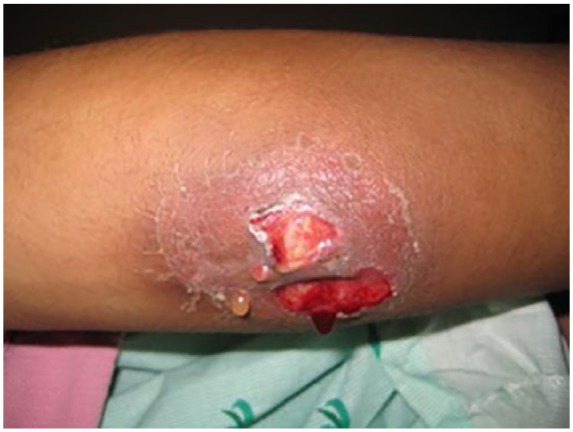
Left elbow on admission.

**Figure 3.**
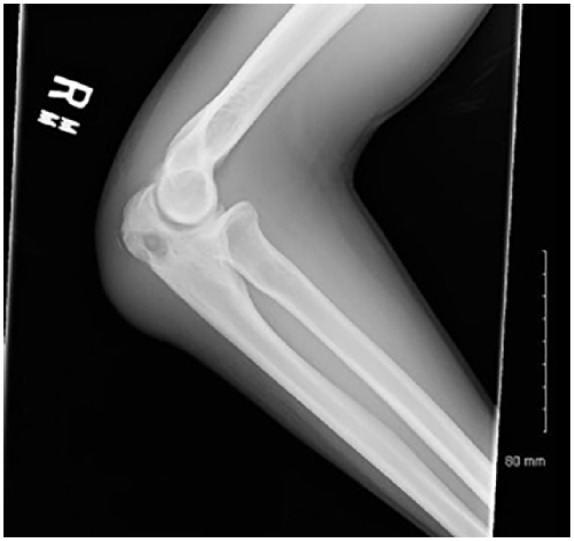
Right elbow X-ray on admission.

**Figure 4.**
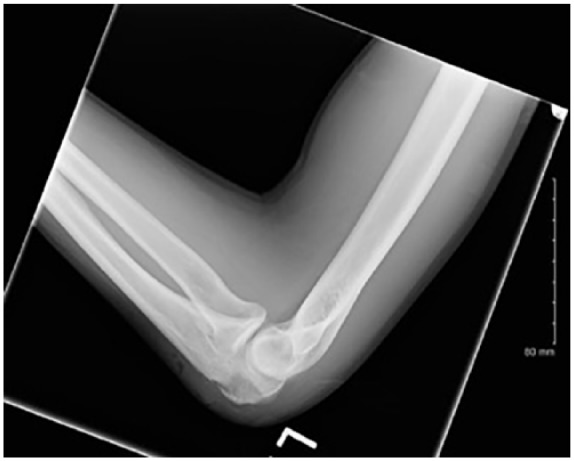
Left elbow X-ray on admission.

**Figure 5.**
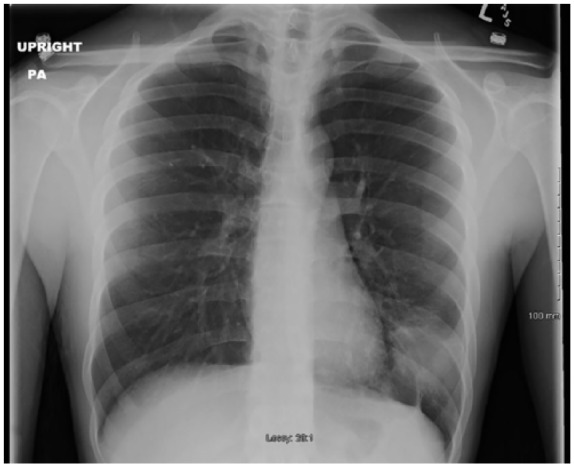
Chest X-ray on admission for case 1.

**Figure 6.**
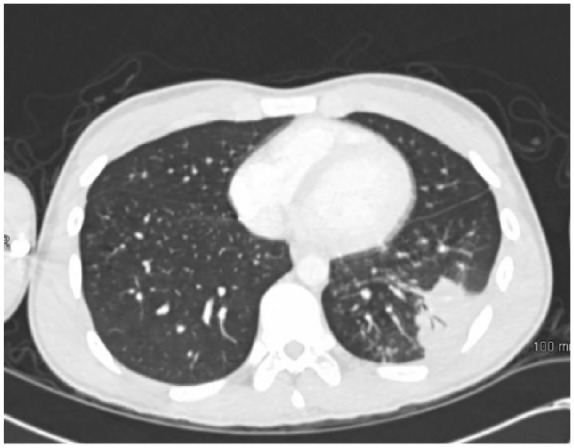
Chest computed tomography on admission for case 1.

The patient started practicing karate in order to improve his energy level. Approximately 2 months PTA, he traumatized both of his elbows during practice, without any apparent integumentary disruption. He noted painful edematous elbows over the next 2 weeks without any improvement. He was seen in the emergency department diagnosed with bilateral elbow bursitis and sent home on oral antibiotics. On the day of admission, without any improvement he noticed skin breaks over both elbows with purulent discharge bilaterally (Figures 1 and 2). Initial imaging revealed bilateral osteomyelitis of both olecranon (Figures 3 and 4). This was confirmed with whole body scan during his hospitalization. He was also found to have a large left lower lobe infiltrate (Figures 5 and 6). Culture of incision and drainage of both elbows demonstrated *Coccidioides immitis*. Subsequently, histopathology showed spherules with endosporulation. Coccidioidal immunodiffusion, IgM, and IgG were positive with complement fixation titer of 1:256.

He was started on oral 1000 mg fluconazole daily. The treatment was switched to amphoteric B liposomal complex (ABL). His elbow underwent additional osseous debridement and skin grafting. ABL was continued for 4 more weeks and transitioned to oral 1000 mg fluconazole daily. The patient was lost to follow-up, last seen 2 years after admission with well-healed wound and full function of bilateral elbows, cleared chest X-ray with coccidioidal serology titers of 1/32.

## Case Presentation 2

A 43-year-old previously healthy Hispanic male resident of Bakersfield with a subacute cough and no other described symptoms suffered a right anterior tibial injury secondary to a fall from a forklift. At an urgent care, there was no evidence of bony injury or a break in the integument; however, swelling, erythema, and pain were appreciated at the site of injury.

**Figure 7.**
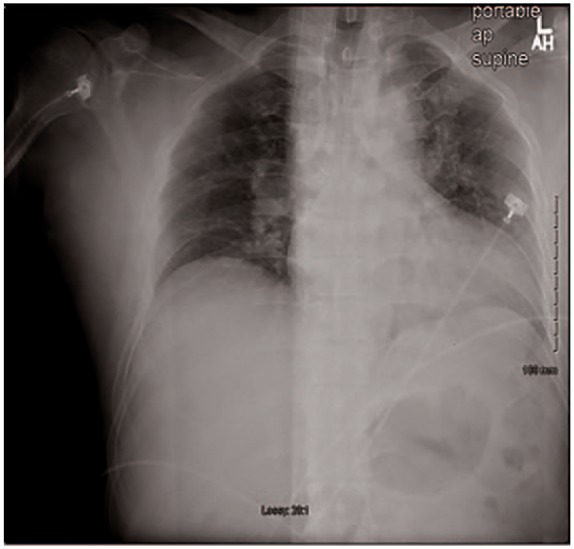
Chest X-ray on admission for case 2.

**Figure 8.**
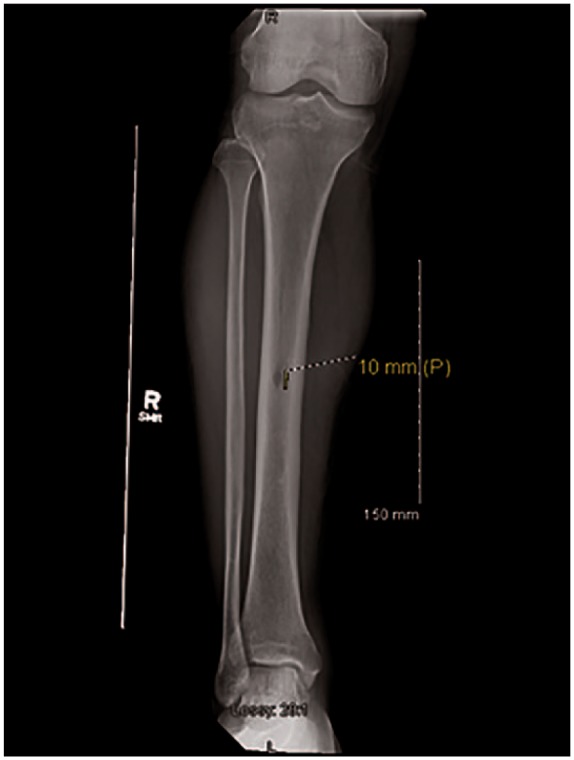
Tibial X-ray on admission for case 2.

Subsequent persistence of swelling, erythema, and pain resulted in a visit to the Kern Medical Emergency Department 30 days after initial trauma. The patient was noted to have cough and subjective fever, as well as pretibial swelling and erythema with no break in the epidermis. Imaging demonstrated right lower lobe pneumonic infiltrate and a lytic lesion in the right tibia (Figures 7 and 8). Operative management included saucerization and debridement. Coccidioidal serology was negative for immunodiffusion IgM and positive for immunodiffusion IgG with a complement fixation titer of 1:32. Intraoperative cultures were positive for *Coccidioides* species. The patient was started on oral 800 mg fluconazole daily. The patient continues to follow-up in clinic with full resolution of the tibial wound and last complement fixation titer of 1:8.

## Discussion

Locus minoris resistentiae is well described in many disease processes including hepatocarcinoma on a cirrhotic liver, the onset of lung carcinoma in tuberculosis scar, and osteosarcoma in the presence of chronic osteomyelitis.^7^ However, there is limited literature identifying it in coccidioidal infections. The sparse literature on coccidioidal LMR can be attributed to the lack of awareness leading to its absence as a differential. Additionally, its similar clinical presentation with direct primary cutaneous coccidioidomycosis further adds to the missed diagnosis.^10^

Dissemination of coccidioidomycosis only occurs in 1% to 5% of those infected,^11^ with an increased likelihood in immunocompromised (including HIV/AIDS, organ transplantation, immunosuppressive medication, diabetes) patients.^12^ While both patients were previously healthy and immunocompetent, other factors may have played a role. A study performed in San Joaquin Valley, California, demonstrated a 5:1 increase in disseminated disease in male compared with female patients, suggesting that a hormonal or genetic component may be involved.^3,13^ The patient’s ethnic background, more specifically those of Filipino and African decent, is another genetic influence that has also been associated with a higher rate of dissemination. While not fully understood, this increased frequency has been associated with certain human leukocyte antigen alleles.^14^ Additionally, it is important to discuss that both patients were residents of the Central Valley California, a highly endemic region with both their jobs (field worker and construction worker) requiring significant amount of exposure to aerosolized arthrocondia in the soil.^15^ While occupational exposure or residency in an endemic area are not associated with a higher risk of dissemination, it may explain to why 2 immunocompetent people were initially infected.

Coccidioidal LMR was first extensively discussed by Dr Pappagianis in 1985,^6^ where he outlines cases of localized coccidioidomycosis in the presence of antecedent trauma with and without skin interruption. While some patients present with preceding coccidioidomycosis, in others, localized coccidioidal lesion is the initial presentation.

The presentation can be attributed to temporal and sequential differences between the primary pulmonary infection and trauma. One possibility is the presence of concurrent coccidioidal infection during the time of injury leading to a hematogenous dissemination throughout the body, with subsequent inoculation at the site of the LMR.^6^ Another possibility is a previously deposited coccidioidal infection in the tissue that begins replicating following a new-onset trauma.^6,16,17^ Finally, an old trauma with chronic irritation/inflammation could serve as a nidus for a newly acquired disseminated coccidioidal infection.^6,18^

In both cases, the patients had a history of pulmonary disease prior to their blunt traumatic injury. On presentation, clinical symptoms and imaging revealed active primary pulmonary coccidioidomycosis, supporting the theory of hematogenous dissemination with consequent localization at the site of the injury. Culture from both cases demonstrated presence of *Coccidioides* species, excluding a reactive manifestation that can occur in up to 50% of acute pulmonary coccidioidomycosis.^19,20^ Lastly, several factors exclude primary cutaneous coccidioidomycosis, per diagnostic criteria established in 1953 by Wilson et al: there should be a lack of a significant pulmonary disease immediately preceding the lesion, history should suggest a break in the skin at the site of the initial lesion, the lesion should be painless and resemble a chancre, the complement fixation should be negative at first and remain negative for several weeks, and, finally, spontaneous healing of the primary cutaneous lesion should occur within a few weeks unless immunologically compromised.^10,21^ Major inconsistencies in the chronological symptomology of our patient thereby exclude a primary cutaneous coccidioidomycosis.

The mechanism for coccidioidal LMR with antecedent trauma remains largely unknown. One possibility is the immunological dysfunction to a region secondary to lymphedema caused by trauma with its subsequent lymph stasis facilitating infection.^7,22^ Another postulation states that the robust inflammatory neutrophil response, while unable to ingest mature spherules, may be responsible for tissue damage as a result of excessive release of oxidants and proteases.^23,24^ Additionally, prostaglandins from damaged leukocytes at the site of injury have been shown to stimulate the formation of the spherule-endospore phase of *Coccidioides immitis.*^6,25^

## Conclusion

While antecedent trauma resulting in LMR is well described in bacterial and mycobacterium infection,^6,10,26,27^ it is less appreciated in fungal infections including coccidioidomycosis. These aforementioned cases are of few disseminated coccidioidomycosis associated with antecedent trauma. Although uncommon, clinicians should be aware of unexplained disseminated coccidioidomycosis secondary to trauma, especially in the setting of travel history to an endemic region.
